# Supplementary material for: Pretrained protein language models choose between sequence novelty and structural completeness
Source: bioRxiv. 2025 Oct 3:2025.10.01.679905. Preprint. [Version 1] doi: 10.1101/2025.10.01.679905 (PMC12621866; doi:10.1101/2025.10.01.679905)
Supplement: 1 [file NIHPP2025.10.01.679905V1-supplement-1.pdf]

bioRxiv preprint doi: https://doi.org/10.1101/2025.10.01.679905; this version posted October 3, 2025. The copyright holder for this preprint (which was not certified by peer review) is the author/funder, who has granted bioRxiv a license to display the preprint in perpetuity. It is made available under aCC-BY 4.0 International license.

Supporting information

Supplemental Figures

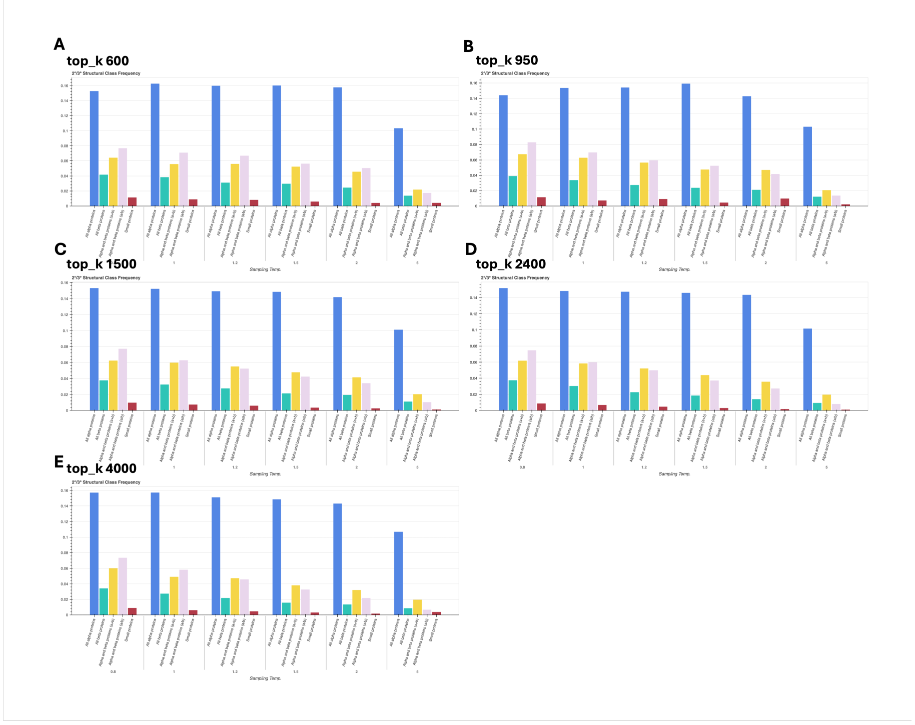

Fig S.1. Structure hit rates from base ProtGPT2 decrease as sampling temperature and top_k increase. Structure hit rates from batches of 100k sequences generated from ProtGPT2 for several sampling temperatures (0.8, 1, 1.2, 1.5, 2, 5) and top_k values (number of highest-probability tokens considered in sampling out of 50,256 total) – (**A**) 600, (**B**) 950, (**C**) 1500, (**D**) 2400, (**E**) 4000; broken down by protein global topology class (α, β, $\alpha + \beta$, α/β, or ”small / minimal 2° structure”)

bioRxiv preprint doi: https://doi.org/10.1101/2025.10.01.679905; this version posted October 3, 2025. The copyright holder for this preprint (which was not certified by peer review) is the author/funder, who has granted bioRxiv a license to display the preprint in perpetuity. It is made available under aCC-BY 4.0 International license.

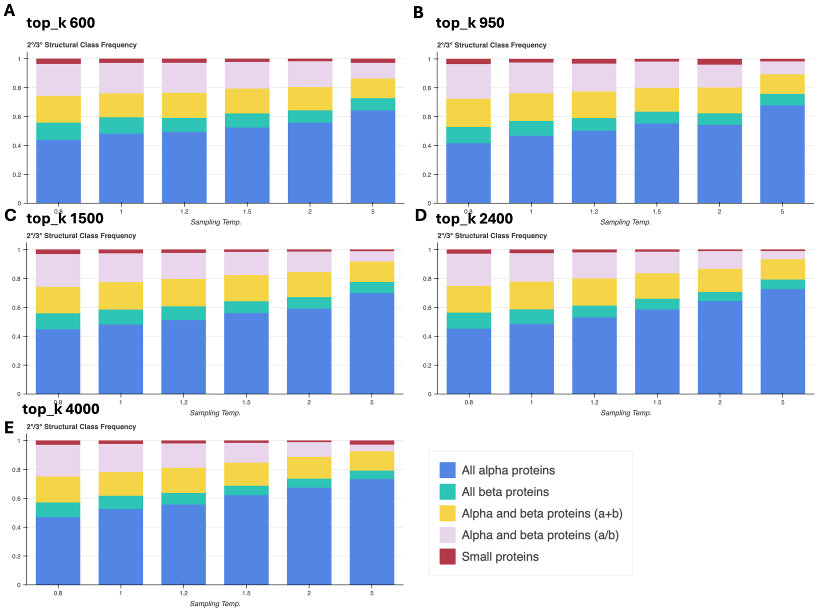

Fig S.2. Generated fold distributions shift towards all-α proteins and away from α/β proteins as sampling temperature increases. Frequency of each protein global topology class (α, β, $\alpha + \beta$, α/β, or "small / minimal 2° structure") among all structure hits within batches of 100k sequences generated from ProtGPT2 for several sampling temperatures (0.8, 1, 1.2, 1.5, 2, 5) and top_k values (number of highest-probability tokens considered in sampling out of 50,256 total) – (**A**) 600, (**B**) 950, (**C**) 1500, (**D**) 2400, (**E**) 4000

bioRxiv preprint doi: https://doi.org/10.1101/2025.10.01.679905; this version posted October 3, 2025. The copyright holder for this preprint (which was not certified by peer review) is the author/funder, who has granted bioRxiv a license to display the preprint in perpetuity. It is made available under aCC-BY 4.0 International license.

Supplemental Tables

Table S.1. Most common SCOP folds generated by base ProtGPT2 at various sampling temperatures with top_k 600.

temp: 0.8

Fold	Class	Freq.	Abs. Hit Rate	Esc. Rate
Long alpha-hairpin	α	0.095	0.033	0.810
Spectrin repeat-like	α	0.050	0.017	0.871
Rossmann(2x3)oid (Flavodoxin-like)	α/β	0.048	0.017	0.146
Immunoglobulin-like beta-sandwich	β	0.036	0.012	0.510
alpha-alpha superhelix	α	0.033	0.011	0.386

temp: 1

Fold	Class	Freq.	Abs. Hit Rate	Esc. Rate
Long alpha-hairpin	α	0.112	0.038	0.874
Spectrin repeat-like	α	0.056	0.019	0.907
Rossmann(2x3)oid (Flavodoxin-like)	α/β	0.043	0.014	0.252
Immunoglobulin-like beta-sandwich	β	0.034	0.012	0.596
Hemerythrin-type up-and-down 4-helical bundle	α	0.032	0.011	0.903

temp: 1.2

Fold	Class	Freq.	Abs. Hit Rate	Esc. Rate
Long alpha-hairpin	α	0.122	0.039	0.908
Spectrin repeat-like	α	0.063	0.020	0.929
Rossmann(2x3)oid (Flavodoxin-like)	α/β	0.043	0.014	0.330
Hemerythrin-type up-and-down 4-helical bundle	α	0.036	0.012	0.937
Immunoglobulin-like beta-sandwich	β	0.030	0.010	0.696

temp: 1.5

Fold	Class	Freq.	Abs. Hit Rate	Esc. Rate
Long alpha-hairpin	α	0.130	0.040	0.943
Spectrin repeat-like	α	0.068	0.021	0.950
Rossmann(2x3)oid (Flavodoxin-like)	α/β	0.042	0.013	0.424
Hemerythrin-type up-and-down 4-helical bundle	α	0.040	0.012	0.958
Immunoglobulin/albumin-binding domain-like	α	0.033	0.010	0.955

temp: 2

Fold	Class	Freq.	Abs. Hit Rate	Esc. Rate
Long alpha-hairpin	α	0.141	0.040	0.969
Spectrin repeat-like	α	0.075	0.021	0.968
Hemerythrin-type up-and-down 4-helical bundle	α	0.044	0.013	0.978
Rossmann(2x3)oid (Flavodoxin-like)	α/β	0.043	0.012	0.500
Immunoglobulin/albumin-binding domain-like	α	0.032	0.009	0.966

temp: 5

Fold	Class	Freq.	Abs. Hit Rate	Esc. Rate
Long alpha-hairpin	α	0.154	0.025	0.989
Spectrin repeat-like	α	0.084	0.014	0.989
Hemerythrin-type up-and-down 4-helical bundle	α	0.060	0.010	0.984
alpha-alpha superhelix	α	0.040	0.006	0.905
Immunoglobulin/albumin-binding domain-like	α	0.033	0.005	0.987

bioRxiv preprint doi: https://doi.org/10.1101/2025.10.01.679905; this version posted October 3, 2025. The copyright holder for this preprint (which was not certified by peer review) is the author/funder, who has granted bioRxiv a license to display the preprint in perpetuity. It is made available under aCC-BY 4.0 International license.

Table S.2. Most common SCOP folds generated by base ProtGPT2 at various sampling temperatures with top_k (vocabulary size) 950.

temp: 0.8				
Fold	Class	Freq.	Abs. Hit Rate	Esc. Rate
Long alpha-hairpin	α	0.101	0.035	0.841
Spectrin repeat-like	α	0.050	0.017	0.872
Rossmann(2x3)oid (Flavodoxin-like)	α/β	0.048	0.016	0.177
Immunoglobulin-like beta-sandwich	β	0.032	0.011	0.534
Canonical WHD (winged helix domain) fold	$\alpha+\beta$	0.031	0.011	0.342
temp: 1				
Fold	Class	Freq.	Abs. Hit Rate	Esc. Rate
Long alpha-hairpin	α	0.111	0.036	0.893
Spectrin repeat-like	α	0.058	0.019	0.918
Rossmann(2x3)oid (Flavodoxin-like)	α/β	0.042	0.014	0.273
Hemerythrin-type up-and-down 4-helical bundle	α	0.034	0.011	0.926
alpha-alpha superhelix	α	0.031	0.010	0.571
temp: 1.2				
Fold	Class	Freq.	Abs. Hit Rate	Esc. Rate
Long alpha-hairpin	α	0.126	0.039	0.930
Spectrin repeat-like	α	0.065	0.020	0.946
Rossmann(2x3)oid (Flavodoxin-like)	α/β	0.041	0.013	0.345
Hemerythrin-type up-and-down 4-helical bundle	α	0.038	0.012	0.948
Canonical WHD (winged helix domain) fold	$\alpha+\beta$	0.030	0.009	0.530
temp: 1.5				
Fold	Class	Freq.	Abs. Hit Rate	Esc. Rate
Long alpha-hairpin	α	0.136	0.039	0.943
Spectrin repeat-like	α	0.069	0.020	0.969
Hemerythrin-type up-and-down 4-helical bundle	α	0.046	0.013	0.960
Rossmann(2x3)oid (Flavodoxin-like)	α/β	0.042	0.012	0.491
Immunoglobulin/albumin-binding domain-like	α	0.030	0.009	0.960
temp: 2				
Fold	Class	Freq.	Abs. Hit Rate	Esc. Rate
Long alpha-hairpin	α	0.149	0.039	0.978
Spectrin repeat-like	α	0.076	0.020	0.984
Hemerythrin-type up-and-down 4-helical bundle	α	0.045	0.012	0.976
Rossmann(2x3)oid (Flavodoxin-like)	α/β	0.040	0.010	0.596
Immunoglobulin/albumin-binding domain-like	α	0.035	0.009	0.974
temp: 5				
Fold	Class	Freq.	Abs. Hit Rate	Esc. Rate
Long alpha-hairpin	α	0.178	0.027	0.991
Spectrin repeat-like	α	0.090	0.014	0.996
Hemerythrin-type up-and-down 4-helical bundle	α	0.064	0.010	0.989
Immunoglobulin/albumin-binding domain-like	α	0.038	0.006	0.986
alpha-alpha superhelix	α	0.035	0.005	0.934

bioRxiv preprint doi: https://doi.org/10.1101/2025.10.01.679905; this version posted October 3, 2025. The copyright holder for this preprint (which was not certified by peer review) is the author/funder, who has granted bioRxiv a license to display the preprint in perpetuity. It is made available under aCC-BY 4.0 International license.

Table S.3. Most common SCOP folds generated by base ProtGPT2 at various sampling temperatures with top_k (vocabulary size) 1500.

Fold	Class	Freq.	Abs. Hit Rate	Esc. Rate
**temp: 0.8**				
Long alpha-hairpin	α	0.101	0.035	0.847
Spectrin repeat-like	α	0.052	0.018	0.878
Rossmann(2x3)oid (Flavodoxin-like)	α/β	0.045	0.015	0.210
Immunoglobulin-like beta-sandwich	β	0.033	0.011	0.555
alpha-alpha superhelix	α	0.031	0.010	0.426
**temp: 1**				
Long alpha-hairpin	α	0.119	0.037	0.895
Spectrin repeat-like	α	0.059	0.019	0.918
Rossmann(2x3)oid (Flavodoxin-like)	α/β	0.040	0.013	0.304
Hemerythrin-type up-and-down 4-helical bundle	α	0.035	0.011	0.930
Canonical WHD (winged helix domain) fold	$\alpha+\beta$	0.029	0.009	0.472
**temp: 1.2**				
Long alpha-hairpin	α	0.129	0.038	0.930
Spectrin repeat-like	α	0.067	0.019	0.956
Rossmann(2x3)oid (Flavodoxin-like)	α/β	0.042	0.012	0.425
Hemerythrin-type up-and-down 4-helical bundle	α	0.040	0.012	0.951
Canonical WHD (winged helix domain) fold	$\alpha+\beta$	0.029	0.008	0.528
**temp: 1.5**				
Long alpha-hairpin	α	0.145	0.038	0.963
Spectrin repeat-like	α	0.077	0.020	0.984
Hemerythrin-type up-and-down 4-helical bundle	α	0.047	0.012	0.976
Rossmann(2x3)oid (Flavodoxin-like)	α/β	0.040	0.011	0.566
Immunoglobulin/albumin-binding domain-like	α	0.033	0.009	0.968
**temp: 2**				
Long alpha-hairpin	α	0.158	0.038	0.988
Spectrin repeat-like	α	0.078	0.019	0.989
Hemerythrin-type up-and-down 4-helical bundle	α	0.050	0.012	0.986
Rossmann(2x3)oid (Flavodoxin-like)	α/β	0.039	0.009	0.708
Immunoglobulin/albumin-binding domain-like	α	0.034	0.008	0.987
**temp: 5**				
Long alpha-hairpin	α	0.182	0.026	0.993
Spectrin repeat-like	α	0.094	0.014	0.999
Hemerythrin-type up-and-down 4-helical bundle	α	0.070	0.010	0.994
Ferredoxin-like	$\alpha+\beta$	0.040	0.006	0.984
Immunoglobulin/albumin-binding domain-like	α	0.038	0.005	0.993

bioRxiv preprint doi: https://doi.org/10.1101/2025.10.01.679905; this version posted October 3, 2025. The copyright holder for this preprint (which was not certified by peer review) is the author/funder, who has granted bioRxiv a license to display the preprint in perpetuity. It is made available under aCC-BY 4.0 International license.

Table S.4. Most common SCOP folds generated by base ProtGPT2 at various sampling temperatures with top_k (vocabulary size) 2400.

Fold	Class	Freq.	Abs. Hit Rate	Esc. Rate
**temp: 0.8**				
Long alpha-hairpin	α	0.106	0.036	0.850
Spectrin repeat-like	α	0.052	0.018	0.894
Rossmann(2x3)oid (Flavodoxin-like)	α/β	0.043	0.014	0.210
alpha-alpha superhelix	α	0.032	0.011	0.435
Canonical WHD (winged helix domain) fold	$\alpha+\beta$	0.031	0.011	0.358
**temp: 1**				
Long alpha-hairpin	α	0.125	0.038	0.905
Spectrin repeat-like	α	0.062	0.019	0.939
Rossmann(2x3)oid (Flavodoxin-like)	α/β	0.040	0.012	0.353
Hemerythrin-type up-and-down 4-helical bundle	α	0.038	0.011	0.917
Canonical WHD (winged helix domain) fold	$\alpha+\beta$	0.028	0.008	0.446
**temp: 1.2**				
Long alpha-hairpin	α	0.138	0.038	0.945
Spectrin repeat-like	α	0.071	0.020	0.959
Hemerythrin-type up-and-down 4-helical bundle	α	0.043	0.012	0.957
Rossmann(2x3)oid (Flavodoxin-like)	α/β	0.041	0.011	0.456
Ferredoxin-like	$\alpha+\beta$	0.030	0.008	0.792
**temp: 1.5**				
Long alpha-hairpin	α	0.158	0.039	0.976
Spectrin repeat-like	α	0.077	0.019	0.985
Hemerythrin-type up-and-down 4-helical bundle	α	0.052	0.013	0.981
Rossmann(2x3)oid (Flavodoxin-like)	α/β	0.038	0.010	0.601
Ferredoxin-like	$\alpha+\beta$	0.033	0.008	0.888
**temp: 2**				
Long alpha-hairpin	α	0.167	0.037	0.994
Spectrin repeat-like	α	0.086	0.019	0.992
Hemerythrin-type up-and-down 4-helical bundle	α	0.056	0.012	0.991
Immunoglobulin/albumin-binding domain-like	α	0.041	0.009	0.991
Ferredoxin-like	$\alpha+\beta$	0.036	0.008	0.956
**temp: 5**				
Long alpha-hairpin	α	0.190	0.027	0.998
Spectrin repeat-like	α	0.095	0.013	0.998
Hemerythrin-type up-and-down 4-helical bundle	α	0.069	0.010	0.998
Ferredoxin-like	$\alpha+\beta$	0.041	0.006	0.993
Immunoglobulin/albumin-binding domain-like	α	0.041	0.006	0.996

bioRxiv preprint doi: https://doi.org/10.1101/2025.10.01.679905; this version posted October 3, 2025. The copyright holder for this preprint (which was not certified by peer review) is the author/funder, who has granted bioRxiv a license to display the preprint in perpetuity. It is made available under aCC-BY 4.0 International license.

Table S.5. Most common SCOP folds generated by base ProtGPT2 at various sampling temperatures with top_k (vocabulary size) 4000.

temp: 0.8				
Fold	Class	Freq.	Abs. Hit Rate	Esc. Rate
Long alpha-hairpin	α	0.108	0.036	0.855
Spectrin repeat-like	α	0.054	0.018	0.892
Rossmann(2x3)oid (Flavodoxin-like)	α/β	0.042	0.014	0.217
alpha-alpha superhelix	α	0.031	0.010	0.448
Hemerythrin-type up-and-down 4-helical bundle	α	0.031	0.010	0.896
**temp: 1**				
Fold	Class	Freq.	Abs. Hit Rate	Esc. Rate
Long alpha-hairpin	α	0.123	0.037	0.904
Spectrin repeat-like	α	0.065	0.019	0.939
Rossmann(2x3)oid (Flavodoxin-like)	α/β	0.039	0.012	0.377
Hemerythrin-type up-and-down 4-helical bundle	α	0.038	0.011	0.930
alpha-alpha superhelix	α	0.028	0.008	0.609
**temp: 1.2**				
Fold	Class	Freq.	Abs. Hit Rate	Esc. Rate
Long alpha-hairpin	α	0.146	0.039	0.949
Spectrin repeat-like	α	0.071	0.019	0.974
Hemerythrin-type up-and-down 4-helical bundle	α	0.046	0.012	0.967
Rossmann(2x3)oid (Flavodoxin-like)	α/β	0.041	0.011	0.544
Ferredoxin-like	$\alpha+\beta$	0.031	0.008	0.812
**temp: 1.5**				
Fold	Class	Freq.	Abs. Hit Rate	Esc. Rate
Long alpha-hairpin	α	0.161	0.038	0.981
Spectrin repeat-like	α	0.086	0.020	0.991
Hemerythrin-type up-and-down 4-helical bundle	α	0.054	0.013	0.982
Immunoglobulin/albumin-binding domain-like	α	0.039	0.009	0.983
Rossmann(2x3)oid (Flavodoxin-like)	α/β	0.035	0.008	0.699
**temp: 2**				
Fold	Class	Freq.	Abs. Hit Rate	Esc. Rate
Long alpha-hairpin	α	0.183	0.039	0.997
Spectrin repeat-like	α	0.092	0.019	0.994
Hemerythrin-type up-and-down 4-helical bundle	α	0.062	0.013	0.998
Immunoglobulin/albumin-binding domain-like	α	0.038	0.008	0.995
Ferredoxin-like	$\alpha+\beta$	0.038	0.008	0.970
**temp: 5**				
Fold	Class	Freq.	Abs. Hit Rate	Esc. Rate
Long alpha-hairpin	α	0.196	0.029	0.999
Spectrin repeat-like	α	0.097	0.014	1.000
Hemerythrin-type up-and-down 4-helical bundle	α	0.079	0.011	1.000
Ferredoxin-like	$\alpha+\beta$	0.040	0.006	0.998
Immunoglobulin/albumin-binding domain-like	α	0.038	0.005	1.000
